# Diagnostic biomarker potential of plasma small extracellular vesicle RNA enriched for microglial origin in people with Dementia with Lewy bodies

**DOI:** 10.1002/alz70856_098303

**Published:** 2025-12-24

**Authors:** Anto Praveen Rajkumar Rajamani, Fatma Busra Isik, Helen Miranda Knight

**Affiliations:** ^1^ University of Nottingham, Nottingham, Nottinghamshire, United Kingdom; ^2^ Nottinghamshire Healthcare NHS foundation trust, Nottingham, Nottinghamshire, United Kingdom; ^3^ University of Nottingham, Nottingham, nottinghamshire, United Kingdom; ^4^ University of Nottingham, Nottingham, Nottingham, United Kingdom

## Abstract

**Background:**

Accurate distinction between Dementia with Lewy Bodies (DLB) and Alzheimer's Disease (AD) is important because they differ in their prognosis, risk profiles, and treatment. As current radioisotopes‐based imaging biomarkers for DLB are not feasible in many clinical settings, there is an urgent clinical need for identifying blood‐based biomarkers. We have recently developed a prototype plasma small extracellular vesicles (SEV) multiplex RNA assay that can differentiate people with DLB from those with AD with nearly 75% accuracy. We are working on refining the assay and improving its diagnostic accuracy. Chronic microglial activation contributes towards AD pathology. Immunohistochemical, transcriptomic and proteomic studies revealed absence of such chronic neuroinflammation in DLB. Hence, investigating plasma SEV RNA, enriched for microglial origin, may help improving the diagnostic accuracy of our blood‐based biomarker assay for DLB.

**Method:**

We collected plasma samples from people with DLB, AD, and people without cognitive impairment or Parkinson's disease (*N* = 30). We separated SEV from platelet‐free plasma using Izon qEV size exclusion chromatography. We enriched plasma SEV for microglial origin by TMEM119 immunoprecipitation. We confirmed separation and enrichment of SEV using cryo‐transmission electron microscopy, ZetaView nanoparticle tracking analysis and Exoview interferometric reflectance imaging. We extracted RNA from the enriched plasma SEV. We identified DLB‐specific differentially expressed RNA using next‐generation RNA‐sequencing.

**Result:**

We standardised a novel protocol for enriching plasma SEV for microglial origin and demonstrated the feasibility of measuring very low‐input RNA levels from clinically collected blood samples. Our RNA‐Seq data analysis pipeline identified 28 RNA, including *MAPK6*, *IRAK1* and *MIR28*, that were significantly differentially expressed in enriched plasma SEV of people with DLB, when compared to AD, after appropriate false discovery rate corrections at 5%. Functional enrichment analyses of the differentially expressed RNA revealed downregulation of Galactosyltransferase activity and several inflammation associated pathways.

**Conclusion:**

Neuroinflammation‐focused plasma SEV RNA can help distinguishing DLB from AD accurately. We will develop a bespoke multiplex plasma SEV RNA assay using the identified differentially expressed RNA and will evaluate its diagnostic accuracy in an independent cohort.

